# Airport Traveler Testing Program for SARS-CoV-2 — Alaska, June–November 2020

**DOI:** 10.15585/mmwr.mm7016a2

**Published:** 2021-04-23

**Authors:** Elizabeth C. Ohlsen, Kimberly A. Porter, Eric Mooring, Coleman Cutchins, Anne Zink, Joseph McLaughlin

**Affiliations:** ^1^Alaska Department of Health and Social Services; ^2^Division of State and Local Readiness, Center for Preparedness and Response, CDC; ^3^Epidemic Intelligence Service, CDC.

Travel can facilitate SARS-CoV-2 introduction. To reduce introduction of SARS-CoV-2 infections, the state of Alaska implemented a program on June 6, 2020, for arriving air, sea, and road travelers that required either molecular testing for SARS-CoV-2, the virus that causes COVID-19, or a 14-day self-quarantine after arrival. The Alaska Department of Health and Social Services (DHSS) used weekly standardized reports submitted by 10 participating Alaska airports to evaluate air traveler choices to undergo testing or self-quarantine, traveler test results, and airport personnel experiences while implementing the program. Among 386,435 air travelers who arrived in Alaska during June 6–November 14, 2020, a total of 184,438 (48%) chose to be tested within 72 hours before arrival, 111,370 (29%) chose to be tested on arrival, and 39,685 (10%) chose to self-quarantine without testing after arrival. An additional 15,112 persons received testing at airport testing sites; these were primarily travelers obtaining a second test 7–14 days after arrival, per state guidance. Of the 126,482 airport tests performed in Alaska, 951 (0.8%) results were positive, or one per 406 arriving travelers. Airport testing program administrators reported that clear communication, preparation, and organization were vital for operational success; challenges included managing travelers’ expectations and ensuring that sufficient personnel and physical space were available to conduct testing. Expected mitigation measures such as vaccination, physical distancing, mask wearing, and avoidance of gatherings after arrival might also help limit postarrival transmission. Posttravel self-quarantine and testing programs might reduce travel-associated SARS-CoV-2 transmission and importation, even without enforcement. Traveler education and community and industry partnerships might help ensure success.

To assess the airport traveler testing program, Alaska DHSS reviewed Alaska’s COVID-19 requirements and testing operations for arriving air travelers during June 6–November 14, 2020. Although travelers entering Alaska by road and sea were also subject to these requirements, entry by road and sea was minimal after Canada began restricting nonessential transit on March 20, 2020 (*1*), and these ports of entry neither provided weekly briefs nor routinely offered onsite testing; therefore, this report is limited to an analysis of the air traveler program. Airport programs were asked to provide weekly reports on the numbers of incoming flights, passengers screened for symptoms, passengers tested within 72 hours before arrival, passengers who chose to self-quarantine for 14 days after arrival, passengers tested at the airport, and positive test results. In addition to comments provided in the weekly briefs, airport program administrators from all 10 participating airports were also asked to provide improvement recommendations; five airports responded in a narrative format, from which themes were extracted. This activity was reviewed by CDC and was conducted consistent with applicable federal law and CDC policy.*

As part of the airport testing program, airports were required to screen travelers arriving from out of state for symptoms, offer testing, and record whether travelers chose testing or self-quarantine. Alaska DHSS contracted with local health organizations and enlisted local governments to staff and manage testing program operations. Program personnel collected samples within or just outside the Transportation Security Administration (TSA) secure area at all 10 airports. Specimens were analyzed by reverse transcription–polymerase chain reaction at the Alaska State Public Health Laboratories and commercial laboratories. Traveler information was initially collected on paper forms and later via the Alaska Travel Portal (i.e., COVIDSECURE), a web-based application created to manage travel-associated COVID-19 data^†^. The software allowed travelers to report symptoms, close contacts, and demographic information and to upload and view test results and enter their self-quarantine location.

A travel mandate implemented in Alaska during March 2020 required all travelers entering Alaska to self-quarantine for 14 days after arrival. In June, testing was introduced as an option to shorten the 14-day quarantine, with a test near the time of arrival and a second test 7–14 days after arrival. In August, the option for a 14-day self-quarantine without testing was removed for nonresidents; testing before travel was encouraged for nonresidents, who were charged a $250 fee if they chose to test at the airport on arrival. Starting in October, the requirement for a second test 7–14 days after arrival was removed (Box).

BOX **Alaska travel mandate and subsequent updates for arriving travelers during the COVID-19 pandemic — Alaska, March 25–November 15, 2020**

**Original mandate (March 25, 2020)** *

- Alaska residents and nonresidents: self-quarantine^†^ for 14 days after arrival in Alaska and monitor for illness
- Critical infrastructure workers: may follow a workforce and community protection plan that outlines alternative strategies to reduce the risk for importation and has been submitted by the traveler’s employer to and approved by the State of Alaska

**First update (June 6, 2020)** **^§^**

Resident and nonresident options

- 14-day self-quarantine after arrival in Alaska
- Test on arrival (free at airport) and 7–14 days later, self-quarantine until receipt of the first result, and minimize interactions^¶^ before the second result
- Arrive with proof of a negative test** within 72 hours of departure
- Arrive with proof of a negative test within 5 days of departure; test on arrival and minimize interactions until second negative test result
  Exceptions to testing and quarantine requirements
- Children aged <2 years
- Critical infrastructure workers following an approved employer plan
- Proof of positive SARS-CoV-2 test >3 weeks before travel and asymptomatic on arrival

**Second update (August 11, 2020)^††^**

Resident options

- 14-day self-quarantine
- Negative test <72 hours before arrival or on arrival and test 7–14 days later (free arrival test, with voucher for free follow-up second test), self-quarantining until first result, and following strict physical distancing^§§^ measures before second result

Nonresident options

- Negative test <72 hours before arrival or on arrival and second test 7–14 days later ($250 for first arrival test, with voucher for free follow-up second test for travelers staying in Alaska for ≥7 days), self-quarantining until first result, and observing strict physical distancing measures before second result
- No self-quarantine option for nonresidents

Exceptions to testing and quarantine requirements

- Children aged <10 years, although they must otherwise follow the same arrival plan followed by the adults with whom they are traveling
- Critical infrastructure workers following an approved employer plan
- Alaska residents leaving the state for <24 hours
- Previous positive molecular SARS-CoV-2 test <90 days before arrival, if asymptomatic and carrying a letter of recovery from a medical provider or public health official

**Third update (October 15, 2020)^¶¶^**

Resident options

- 14-day self-quarantine
- Negative test <72 hours before arrival or on arrival (free test, with second test recommended but not required 5–14 days after arrival), self-quarantining until the first result and observing strict physical distancing measures for 5 days after arrival

Nonresident options on arrival

- Negative test <72 hours before arrival or on arrival ($250 test, with second test recommended but not required 5–14 days after arrival), self-quarantining until the first result and observing strict physical distancing measures for 5 days

Exceptions to testing and quarantine requirements

- Children aged <10 years, although they must otherwise follow the same arrival plan followed by the adults with whom they are traveling
- Critical infrastructure workers following an approved company plan
- Alaska residents leaving the state for <72 hours
- Previous positive molecular SARS-CoV-2 test <90 days before arrival, if asymptomatic and carrying a letter of recovery from a medical provider or public health official

Fourth update (November 15, 2020)***

• Additional requirement that arriving travelers file a self-isolation plan in case they receive a positive test result on arrival.

* https://content.govdelivery.com/accounts/AKDHSS/bulletins/282d20b

^†^ Self-quarantine is only required at the final destination. Travelers may not leave their quarantine location or be <6 feet from others except to seek medical care. Travelers must monitor for symptoms daily and be tested immediately if symptoms develop.

^§^ https://www.adn.com/alaska-news/2020/06/03/read-the-full-text-of-alaskas-updated-health-mandate-on-interstate-and-international-travel/

^¶^ Travelers must wear face masks in public places, must avoid gatherings and indoor venues, and may not dine inside restaurants.

** Molecular tests, including reverse transcription–polymerase chain reaction tests, were accepted for arriving travelers; antigen tests were not. Travelers were allowed to provide proof of a pending test result and were required to self-quarantine until the result of the first test was available.

^††^ http://www.dot.state.ak.us/faiiap/pdfs/MANDATE-010-Alaska-Travel.pdf

^§§^ Travelers may not enter indoor public spaces, participate in group activities, or attend gatherings. They may be in outdoor public spaces but must remain >6 ft from others and wear a mask at all times.

^¶¶^ https://covid19.alaska.gov/wp-content/uploads/2020/10/10152020-COVID-MANDATE-010-REVISED.pdf

*** https://covid19.alaska.gov/wp-content/uploads/2020/11/Outbreak-Health-Order-No-6-lnternational-and-Interstate-Travel.pdf

During June 6–November 14, 2020, a total of 386,435 air travelers who arrived in Alaska were screened for symptoms; 184,438 (48%) arrived with proof of a negative or pending SARS-CoV-2 test result, 111,370 (29%) chose to be tested on arrival, and 39,685 (10%) chose to self-quarantine after arrival for 14 days without testing (Figure 1). The remaining 50,942 (13%) travelers were exempt from the testing and quarantine requirements because they 1) were following an alternative workforce protection plan outlining alternative strategies to reduce the risk for importation that had been submitted by their employer to the state, 2) arrived with a previous positive test result and proof of completion of isolation, 3) had traveled outside Alaska for <72 hours, 4) left the airport before screening, or 5) were a child exempt from screening requirements because of age. Weekly airport briefs submitted to Alaska DHHS indicated that <10 travelers each week were noncompliant with registration or screening. An additional 15,112 persons received testing at airport testing sites; these were primarily travelers obtaining a second test 7–14 days after arrival, per state guidance.

**FIGURE 1:**
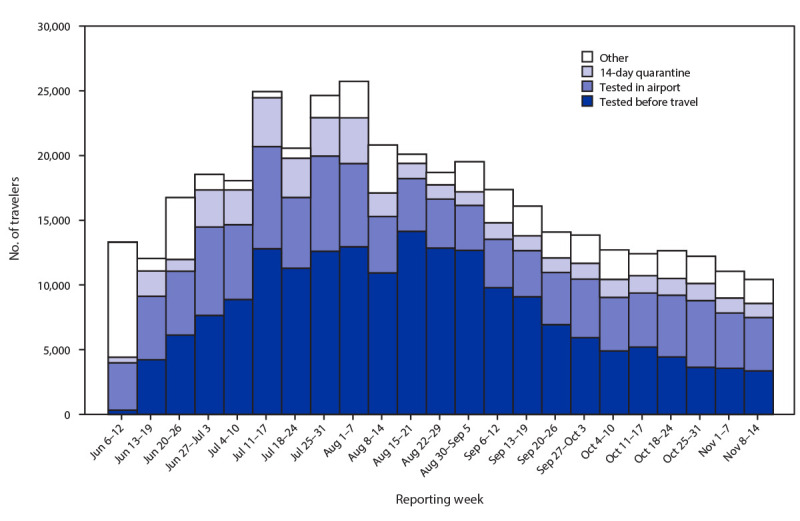
Number of air travelers* who chose self-quarantine after arrival or SARS-CoV-2 testing before travel or at airport on arrival,^†^ by date^§^ — 10 airports, Alaska, June 6–November 14, 2020^¶^ * Paper forms used by certain airports before August 15, 2020, allowed some travelers to select multiple options. ^†^ The travel mandate required two tests (one near the time of arrival and a second test 7–14 days after arrival); the first test date for those tested in the airport is shown, calculated by subtracting the number of second-test vouchers redeemed for airport testing from the total number of travelers tested. ^§^ On August 29, 2020, airport programs switched from reporting data on a Saturday–Friday schedule to a Sunday–Saturday schedule, resulting in an 8-day report for that week. ^¶^ “Other” includes children aged <2 years (exempt from testing), critical infrastructure workers following an alternative workforce and community protection plan, and travelers who arrived with proof of 1) a positive test result within the past 90 days and 2) completion of isolation. Beginning August 11, 2020, children aged <10 years were also exempt from testing.

During June–September, <1.0% of airport test results were positive; this increased to 2.6% during October–November (Figure 2). Over the entire study period (June–November), 951 tests were positive (0.8% overall). The percentage of test results that were positive at airports was consistently lower than the overall percentage in Alaska.

**FIGURE 2:**
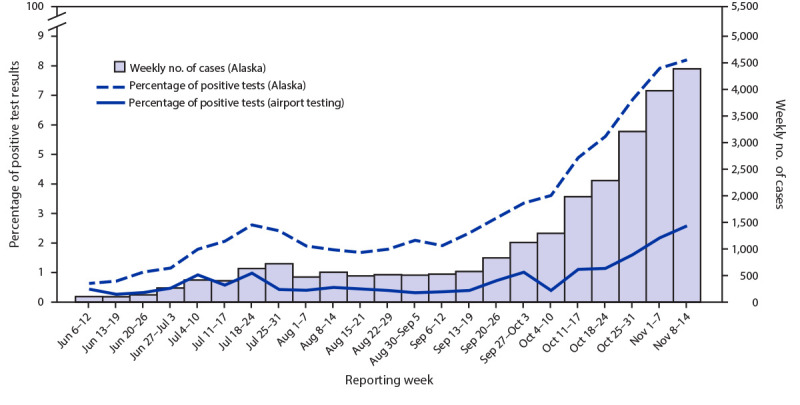
Percentage of positive SARS-CoV-2 test results among air travelers arriving from out of state, percentage of positive SARS-CoV-2 test results statewide, and weekly number of SARS-CoV-2 cases statewide, by specimen collection date* — 10 airports, Alaska, June 6–November 14, 2020 * When specimen collection date was not available, the report date, date of hospitalization, or date of symptom onset was used, whichever was earliest.

In response to a November survey, airport testing program administrators reported that clear communication, preparation, and organization were important for operational success; challenges included managing travelers’ expectations and ensuring sufficient personnel and physical space. For example, administrators reported that travelers were frequently unprepared for screening and that space limitations resulted in travelers being unable to maintain sufficient physical distance. One airport noted an improvement in passenger attitudes and their willingness to complete declaration forms after the initiation of a broad educational campaign for travelers, a hotline for travelers to ask questions, and targeted messaging for travelers before and during travel. Administrators also reported that the travel screening and testing program was resource-intensive. For example, during June–November, Alaska’s largest airport had a weekly average of nearly 12,000 passengers and 51 out-of-state flight arrivals; this airport required up to 22 screening personnel and five testing personnel per day and performed an average of approximately 3,500 tests per week. The cost of this program was also substantial, with a budget of $26 million for June–December.

## Discussion

The primary goal of Alaska’s airport traveler testing program is to reduce the number of travel-associated SARS-CoV-2 importations into the state. During June–November 2020, the program identified 951 persons with a positive SARS-CoV-2 test result Although the number of persons who were infectious during or after travel is unknown, detection and isolation of these travelers likely helped reduce secondary transmission within Alaska. The percentage of positive airport test results remained very low (<1.0%) until October, when it began increasing along with increasing COVID-19 incidence nationwide (*2*). The testing program detected one case per 406 arriving travelers, more than might be expected from symptom screening alone (*3*). Pretravel testing was encouraged, and approximately one half of all arriving travelers were tested before travel. This volume of pretravel testing likely also resulted in some travelers choosing to postpone travel after receiving a positive result, although changes in travel plans were not tracked through this program. Expected mitigation measures such as vaccination, physical distancing, mask wearing, and avoidance of gatherings after arrival might also help limit postarrival transmission. Posttravel self-quarantine and testing programs might reduce travel-associated SARS-CoV-2 transmission and importation, even without enforcement. Traveler education and community and industry partnerships might help ensure success.

Implementation of the traveler testing program required considerable financial and human resources. Funding was attained primarily through the Epidemiology and Laboratory Capacity federal grant and Federal Emergency Management Agency reimbursement. Employing local community contractors, local emergency medical services personnel, and available tourism and hospitality workers helped mitigate the workload for public health personnel. Nonresidents who received positive test results on arrival or who traveled with a pending test that was later reported as positive were often difficult to contact, a problem also encountered in other jurisdictions (*4*). Moreover, contact tracing required extensive interjurisdictional coordination with local, state, Tribal, and federal public health partners. Additional public health resources were also required to address housing challenges for travelers requiring isolation or quarantine.

Traveler education and local community and industry partnerships were critical for successful operations. These efforts resulted in a very low number of travelers evading arrival registration, although the program was not enforced. In partnership with the Alaska Travel Industry Association, using workers to educate passengers about travel requirements before travel was helpful in ensuring passenger compliance with online registration and testing. Alaska’s travel guidance encouraged testing before travel, and nonresident travelers were required to pay $250 for postarrival testing beginning in August; both factors might have increased pretravel testing among nonresidents and likely led to fewer arrivals of infected travelers than might have otherwise occurred.

The findings in this report are subject to at least six limitations. First, test result data were derived from airport testing program briefs and could not be independently verified against laboratory results. Second, handwritten travel declarations used before implementation of an electronic system resulted in some passengers checking multiple options or providing illegible information. Third, test collection sites were outside of TSA secure areas; therefore, a small number of community members might have used airport testing when they had not traveled and were misclassified as travelers. Fourth, participation in screening on arrival was not enforced and a small number of arriving travelers did not complete screening or select testing or self-quarantine. Fifth, the travel program did not include mechanisms for enforcement or for tracking of traveler point of origin, residency, or purpose of travel. In addition, the program relied on existing contact tracing systems for management of positive test results and did not include monitoring of road or seaports of entry. Finally, comprehensive data on postarrival testing or on compliance with movement or activity restrictions were not collected, and data were not available on prospective travelers who changed pretravel plans because of a positive pretravel test.

Based on feedback from Alaska airport testing program administrators, educating travelers on jurisdictional travel requirements before and during travel was helpful. Requiring travelers to have a negative test result within 72 hours before travel could reduce resource requirements for public health services in the arriving location; however, combining this requirement with a postarrival self-quarantine could be considered, because pretravel testing might be less effective than testing after arrival if used as a sole strategy. At least one model suggests that testing within 24 hours before travel would substantially decrease transmission at the destination compared with a test 72 hours before travel.**^§^**

Additional strategies that were helpful in implementing the program included 1) ensuring that passengers were familiar with travel requirements before travel, 2) creating sufficient physical space at airports for efficient testing throughput, 3) offering ready assistance for arriving travelers at airports, and 4) using an electronic traveler interface to notify passengers of their test results, provide information on travel requirements, and collect information on each traveler’s point of origin and travel plans. A traveler data system that coordinates with surveillance systems might reduce the administrative workload on public health officials.

Detecting nearly 1,000 cases of COVID-19 among arriving travelers likely reduced onward transmission from these persons. Likewise, pretravel testing likely prevented many imported cases. Although the impact of Alaska’s traveler testing program on the course of the COVID-19 pandemic in Alaska cannot be quantified with the available data, infectious disease models suggest that reducing the number of imported cases likely delays the occurrence of the peak of an epidemic (*5*), which in turn affords more time to increase public health and health care capacity. Expected mitigation measures such as vaccination, physical distancing, mask wearing, and avoidance of gatherings after arrival might also help limit postarrival transmission.

**Summary**

**What is already known about this topic?**

To reduce traveler-related introduction of SARS-CoV-2 into Alaska, the state instituted a traveler testing program in June 2020. Travelers could be tested within 72 hours before arrival or on arrival or could quarantine for 14 days without testing.

**What is added by this report?**

SARS-CoV-2 testing on arrival in Alaska airports identified 951 SARS-CoV-2 infections, or one per 406 arriving travelers, and might have contributed to Alaska’s low incidence during the summer by reducing opportunities for community transmission at travelers’ destination locations.

**What are the implications for public health practice?**

Posttravel self-quarantine and testing programs might reduce travel-associated SARS-CoV-2 transmission and importation, even without enforcement. Traveler education and community and industry partnerships might help ensure success.

## Footnotes

* 45 C.F.R. part 46.102(l)(2), 21 C.F.R. part 56; 42 U.S.C. Sect. 241(d); 5 U.S.C. Sect. 552a; 44 U.S.C. Sect. 3501 et seq.

† https://covidsecureapp.com/index.html

§
https://www.medrxiv.org/content/10.1101/2020.11.23.20237412v1
